# Is There Another Posterior Approach for Presacral Tumors Besides the Kraske Procedure? — A Study on the Feasibility and Safety of Surgical Resection of Primary Presacral Tumors *via* Transsacrococcygeal Transverse Incision

**DOI:** 10.3389/fonc.2022.892027

**Published:** 2022-05-26

**Authors:** Xudong Zhao, Sixin Zhou, Na Liu, Peiyu Li, Lin Chen

**Affiliations:** Senior Department of General Surgery, the First Medical Center of Chinese People’s Liberation Army (PLA) General Hospital, Beijing, China

**Keywords:** prescral tumors, surgery, recurrence, prognosis, transsacrococcygeal transverse incision

## Abstract

**Background:**

The aim of the present study was to explore the feasibility and safety of the surgical resection of presacral tumors *via* a transsacrococcygeal transverse incision.

**Methods:**

The clinical data and prognoses of patients with presacral tumors who underwent surgery at the Chinese People’s Liberation Army (PLA) General Hospital between January 2009 and December 2018 were retrospectively reviewed and analyzed.

**Results:**

A total of 110 patients with presacral tumors were included in this study, including 82 female patients and 28 male patients, with a female-to-male ratio of 2.9:1. A posterior approach (transsacrococcygeal transverse incision) was utilized in 105 patients, an anterior approach (transabdominal excision) was utilized in 1 patient, and a combined (posterior plus anterior) approach was utilized in 4 patients. The mean tumor size was 8.72 ± 4.28 cm. More than half of the patients (n=59/110) with presacral tumors were asymptomatic. Twenty-six pathological types were observed in our study, including 97 benign lesions and 13 malignant lesions. The intraoperative complication rate was 42.7% (n=47/110), whereas the postoperative morbidity rate was 3.6% (n=4/110). The length of hospital stay for patients treated with the posterior approach was shorter than that of patients treated with the anterior and combined approaches. After a mean follow-up of 90.13 ± 31.22 months, 11 patients had local presacral tumor recurrence, and 1 patient had distant metastasis, with a combined recurrence rate of 10.9% (n=12/110).

**Conclusions:**

The surgical resection of primary presacral tumors *via* a transsacrococcygeal transverse incision is feasible and safe, with acceptable oncological therapeutic outcomes and a low postoperative morbidity rate, making it worth popularizing in clinical practice.

## Introduction

The presacral space, which is also known as the retrorectal space, represents a potential space. Presacral tumors originate in the presacral space. Presacral tumors are uncommon in the clinic (1) and arise from multiple histoembryonic origins in the space between the rectum and sacrum. Previous studies have shown an incidence of 1 in 40,000 for these tumors (2). Most presacral tumors are benign, but malignant tumors can also arise in the presacral space (3, 4). Presacral tumors can be congenital or acquired (4).

Complete surgical resection is the gold standard and most effective treatment for presacral tumors (4–6). The presacral space is a potential space with complex adjacent tissue structures, which makes surgery difficult to perform (7). To date, the surgical approaches to resect presacral tumors that have been reported in the literature include anterior (abdominal incision), posterior (sacrococcygeal incision), and combined anterior+posterior approaches, as well as laparoscopic surgery (8, 9). Among most of published studies (7, 10–14), posterior approaches are the most common.

There are many variations of posterior approaches, including the transsphincteric (transrectal) approach, intersphincteric approach (15), transanal approach (16), transsacrococcygeal approach (17), transvaginal approach, and Kraske or modified Kraske approach (10, 15).

For the resection of presacral tumors, most studies reported the use of a posterior approach, mainly the Kraske approach or modified Kraske approach (5, 10, 18). A newly published multicenter French study in Annals of Surgery validated the value of the modified Kraske approach in the surgical resection of presacral tumors (10). However, at our center, we prefer to use a transsacrococcygeal transverse incision for the resection of primary presacral tumors based on the safety of the procedure. After searching the literature, we found no reports about the surgical resection of primary presacral tumors *via* a transsacrococcygeal transverse incision based on the surgical technique and long-term follow-up outcomes in a large sample of patients. In this study, through a retrospective analysis of our previous surgical data, we evaluated the value of the transsacrococcygeal transverse incision in the resection of presacral tumors based on the safety of the surgical technique and long-term prognosis.

## Materials and Methods

### Patients

Patients were identified using the phrase ‘presacral tumor’ or ‘retrorectal tumor’ to search our prospective database of all patients with presacral or retrorectal tumors that were treated at the Chinese People’s Liberation Army (PLA) General Hospital between January 2009 and December 2018. Patients with anal fistulas, pilonidal sinuses, hemorrhoids, perianal abscesses, metastatic presacral tumors, recurrent lesions, or malignancies originating from the rectum or reproductive system or who were less than 18 years old were excluded. Patients who did not undergo surgery were also excluded. The study was approved by the ethics committee of the Chinese PLA General Hospital.

All patients had to undergo abdominal and pelvic computed tomography (CT) scans after admission; when necessary, pelvic magnetic resonance imaging (MRI) was performed. Before surgery, the sacral levels of the superior margin of the presacral tumors were obtained by evaluating the sagittal CT or MRI scans of each patient.

### Main Surgical Procedures

**Figure 1:**
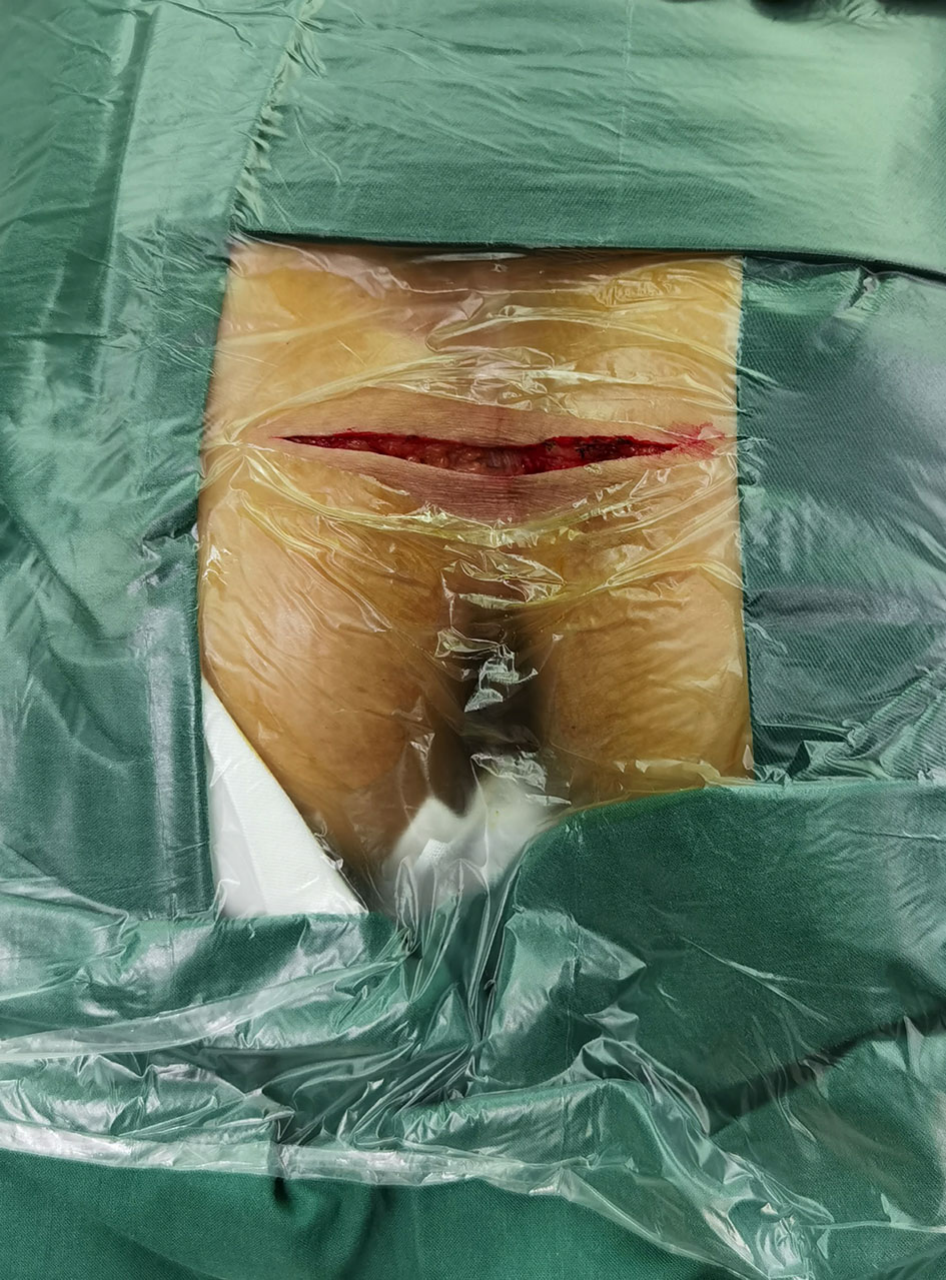
Surgical incision.

**Figure 2:**
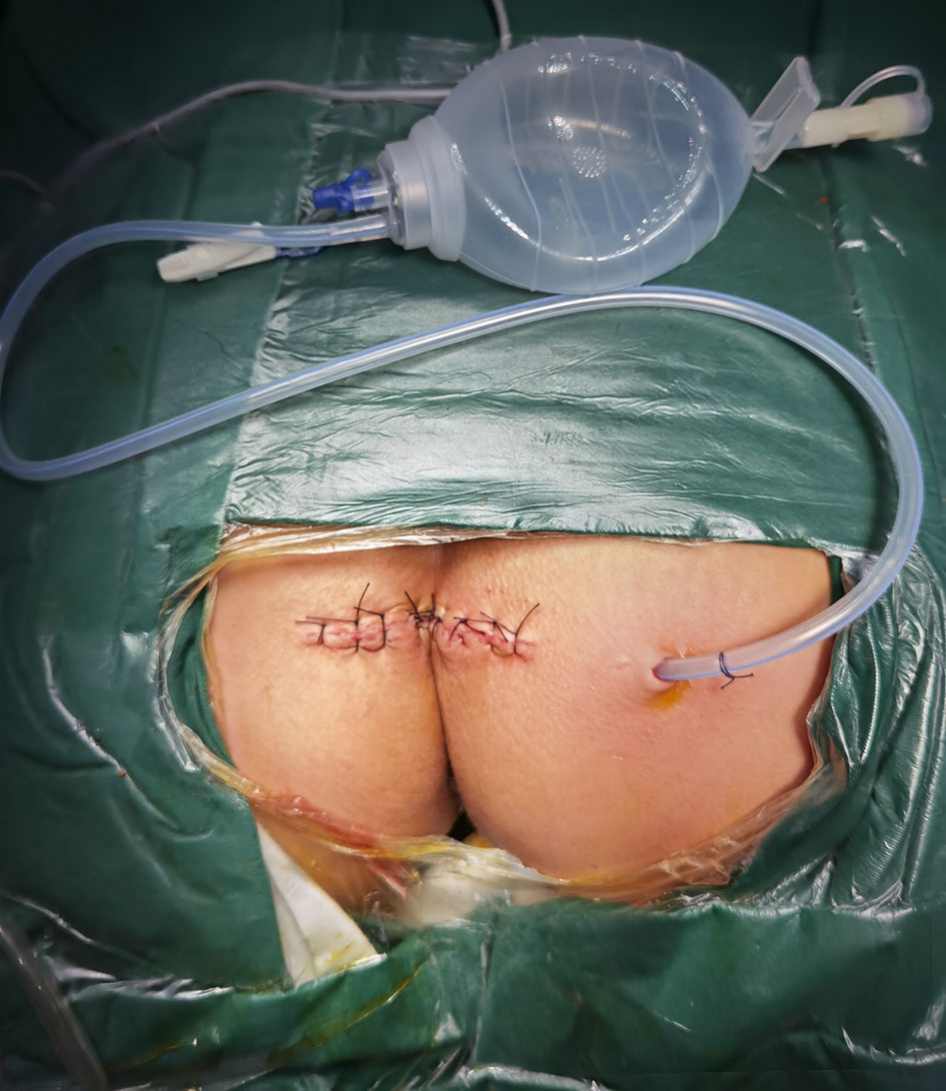
A negative-pressure aspiration device was placed in the surgical site to drain the surgical site after surgery.

After general anesthesia, patients were placed in the prone jack-knife position with the buttocks spread. Then, a transverse incision was made (incision length: approximately 10 cm) one cm below the coccygeal tip. The anococcygeal ligament was cut to obtain access to the presacral space. When necessary, the coccyx was resected. When the tumor was large, vertebrae below the S2 vertebra were resected to expose the lesions. Then, the presacral tumor was meticulously separated from adjacent normal tissues with careful protection of the rectum. When no clear boundary was noted between the presacral tumor and the rectum, rectal palpation with the left hand could be used to indicate the boundary between the presacral tumor and the rectum to better protect the rectum. If rectal injury was unavoidable, small rectal injuries could be resolved by rectal repair. When the rectal injury was large and the patient’s general condition was poor, a prophylactic colostomy was performed. After the tumor was removed, the surgical field was rinsed completely. Then, a drainage tube with a negative-pressure aspiration device was placed in the surgical field. Finally, the surgical incision was sutured (**Figures 1**, **2**).

The operative time was calculated from incision of the skin to suturing of the skin.

Complete surgical resection is the main treatment for presacral tumors and our primary objective. For patients with presacral tumors with superior margins that do not surpass the S1 vertebra, we first consider using a posterior approach for tumor resection. During surgery, if operative difficulties (exposure difficulties, bleeding, etc.) are encountered, the surgical approach is changed from posterior to anterior. In 2017, we attempted to use laparoscopy to resect a presacral tumor. Due to exposure difficulties, the patient underwent conversion from laparoscopy to surgery with a posterior approach.

### Intraoperative Complications and Postoperative Morbidity

According to Aubert M et al. (10), the intraoperative complications mainly include tumor perforation, presacral bleeding, and rectal and bladder perforation. Postoperative morbidity was defined as any surgical or medical complication occurring during the hospital stay or within 30 days after surgery and was mainly represented by wound and pelvic abscesses. Complications were recorded using the Clavien–Dindo classification (19).

### Follow-Up

Follow-up was performed by telephone or outpatient visits as follows: every 3 months in the first year, every six months in the second and third years, and yearly thereafter. A pelvic CT scan was required during follow-up, and when necessary, MRI was added to make a clear diagnosis. The beginning of follow-up was the day when the surgery was performed. The last follow-up time was October 2021.

### Statistics

SPSS 20.0 for Windows (Chicago, IL) was utilized to perform data processing. Numerical data are presented as the mean ± standard deviation (SD), and categorical data are presented as absolute numbers and percentages. Student’s t test was used to compare quantitative data, and Pearson’s chi-squared test was used to compare categorical data. A P value < 0.05 was considered to be statistically significant.

## Results

After searching our prospective database, 187 patients with presacral tumors were initially identified. According to the inclusion and exclusion criteria discussed in the Methods section, 110 patients were ultimately included in this study.

In this study, patients with presacral tumors were younger, with a mean age of 40.0 ± 13.2 (range, 12-69) years at diagnosis. In our study, presacral tumors also showed a female predominance; there were 82 female patients and 28 male patients, with a female-to-male ratio of 2.9:1. All patients (n=110) underwent abdominal and pelvic CT scans before surgery; moreover, 40% (n=44) underwent MRI scans. The demographic and clinical data of the study are summarized in **Table 1**.

**Table 1:** Demographic and clinical data of the study.

Items	Number (n = 110) (%)	Average	T test
**Gender**			
Male	28 (25.5%)		
Female	82 (74.5%)		
**Age(years)**		40.0 ± 13.2	
<40	54 (49.1%)		
≧40	56 (50.1%)		
**Modus operandi**			
Posterior approach	105 (95.5%)		
Anterior and combined approach	5 (4.5%)		
**Tumor size(cm)**		8.72 ± 4.28	
<9	64 (58.2%)		
≧9	46 (41.8%)		
**Length of hospital stay**			*P *= 0.056
Posterior approach	105 (95.5%)	13.3 ± 6.14d	
Anterior and combined approach	5 (4.5%)	19.0 ± 11.8d	
**Pathology**			
Benign	97 (88.2%)		
Malignant	13 (11.8%)		
**Body mass index (BMI)**			
<18.5	9 (8.2%)		
18.5-23.9	57 (51.2%)		
24.0-27.9	35 (31.8%)		
≧28.0	9 (8.2%)		
**Adjacent bone resection**			
No	66 (60.0%)		
Coccygectomy	39 (35.5%)		
Coccygectomy plus partial sacrectomy	5 (4.5%)		
**Co-morbidity**			
No	59 (53.6%)		
Intraoperative complications	47 (42.7%)		
Postoperative complications	4 (3.6%)		
**Operative time**			*P* < 0.05
Posterior approach	105 (95.5%)	125.3 ± 69.0	
Anterior and combined approach	5 (4.5%)	254.0 ± 44.8	
**Sacral levels of the superior margin**			
S1 vertebra	6 (5.5%)		
S2 vertebra	6 (5.5%)		
S3 vertebra	12 (10.9%)		
Below S3 vertebra	86 (78.2%)		
**Preoperative workup**			
Computed Tomography (CT)	110 (100%)		
Magnetic Resonance Imaging (MRI)	44 (40.0%)		
Biopsy	9 (8.2%)		

### Symptoms

In our study, more than half of the patients (n=59/110) with presacral tumors were asymptomatic, and the tumors were discovered incidentally during routine health examinations or testing for other diseases. The symptoms due to the presacral tumors varied and are summarized in **Table 2**.

**Table 2:** Symptoms observed in the study.

Symptoms	Case (s) (%)
asymptomatic	59 (53.6%)
sacral caudal pain	16 (14.5%)
difficult defecation	9 (8.2%)
low back pain/discomfort	6 (5.5%)
stomachache	4 (3.6%)
anal pain/discomfort	4 (3.6%)
constipation	3 (2.7%)
changes in bowel habits	3 (2.7%)
dysuria	2 (1.8%)
abdominal distention	2 (1.8%)
frequent urination	1 (0.9%)
hematuresis	1 (0.9%)

### Pathology

All patients with presacral tumors underwent gross complete resection, but 13 patients (11.7%, n=13/110) underwent R1 resection (involved resection margin) of both benign (n=8) and malignant (n=5) presacral tumors after pathological confirmation. Among the 5 patients with malignant presacral tumors who underwent R1 resection, 3 patients received adjuvant therapy (chemotherapy=2, radiotherapy=1).

In this study, 110 patients presented 26 pathological types, including 97 benign lesions and 13 malignant lesions. The pathological results are summarized in **Table 3**.

**Table 3:** Pathologies observed in the study.

Pathology	Case (s) (%)
**Benign**	**97 (88.2%)**
epidermoid cyst	37 (33.6%)
mature teratoma	28 (25.5%)
benigh cyst	7 (6.4%)
fibromatosis	5 (4.5%)
bronchogenic cyst	2 (1.8%)
foregut cyst	2 (1.8%)
enterogenous cyst	2 (1.8%)
fibroma	2 (1.8%)
neurogenic tumor	2 (1.8%)
inflammatory mass	2 (1.8%)
neurinoma	2 (1.8%)
tailgut cyst	1 (0.9%)
myopericytoma	1 (0.9%)
lipoma	1 (0.9%)
ganglioneuroma	1 (0.9%)
neurofibromatosis	1 (0.9%)
aggressive angiomyxoma	1 (0.9%)
**Malignant**	**13 (11.8%)**
teratoma with malignant transformation	3 (2.7%)
fibromyxoid sarcoma	2 (1.8%)
carcinosarcoma	1 (0.9%)
gastrointestinal stromal tumor	1 (0.9%)
bronchogenic cyst with malignant transformation	1 (0.9%)
tailgut cyst with malignant transformation	1 (0.9%)
neurofibroma with malignant transformation	1 (0.9%)
adenocarcinoma	1 (0.9%)
liposarcoma	1 (0.9%)
chordoma	1 (0.9%)

### Surgery and Complications

In our study of 110 patients, a posterior approach (via a transsacrococcygeal transverse incision) was utilized in 105 patients, an anterior approach (*via* transabdominal excision) was performed in 1 patient, and a combined approach was used in 4 patients, including 1 patient treated with a laparoscopic-assisted anterior + posterior approach and 3 patients treated with a transabdominal anterior + posterior approach (**Table 4**). The mean operative time for the whole series was 130.5 ± 73.1 (range, 25-575) minutes. The mean operative time for the posterior approach alone was 125.3 ± 69.0 (range, 25-575) minutes. The mean operative time for the anterior and combined approaches was 254.0 ± 44.8 (range, 190-315) minutes. The mean operative time for the posterior approach alone was much shorter than that for the anterior and combined approaches (P<0.05). The mean tumor size for the whole series was 8.72 ± 4.28 (range, 2-30) cm. The mean tumor size for the posterior approach alone was 8.52 ± 3.83 (range, 2-20) cm. The mean tumor size for the anterior and combined approaches was 10.00 ± 3.54 (range, 6-15) cm. The mean tumor size for the anterior and combined approaches was greater than that of the posterior approach alone, but the difference between the two groups was not statistically significant (P=0.50). Among these 110 surgeries, coccygectomy was performed in 39 patients, and coccygectomy plus partial sacrectomy was performed in 5 patients (**Table 1**).

**Table 4:** Clinical data of the five anterior and combined approaches.

No.	Gender	Age(years)	Date of surgery	First symptom	Procedures	Sacral levels of the superior margin	Bleeding(ml)	Pathology	complications	Follow-up(months)	recurrence
1	Female	28	Apr. 2017	changes in bowel habits	From laparoscopy to posterior approach	Below S3	100	epidermoid cyst	Tumor perforation	54	Yes
2	Male	46	Apr. 2010	asymptomatic	From posterior approach to anterior(open) approach	S3	3000	fibroma	Presacral bleeding	138	No
3	Male	47	Sep. 2011	changes in bowel habits	anterior (open) approach	S1	800	epidermoid cyst	Tumor perforation	121	No
4	Male	50	Feb. 2012	low back pain	From posterior approach to anterior(open) approach	S1	800	neurofibromatosis	No	116	No
5	Male	62	Dec. 2015	low back pain	From posterior approach to anterior(open) approach	S1	200	neurogenic tumor	No	70	No

Intraoperative complications occurred in 47 patients. Sigmoidostomy was performed in 1 male patient due to intraoperative rectal injury; transverse colostomy was performed in 1 female patient due to intraoperative rectal injury. During surgery, due to tumor compression and adhesion, 3 female patients experienced vaginal wall damage and underwent repair after the presacral tumor was removed completely, and 5 female patients experienced posterior rectal wall damage during surgery and underwent repair after the presacral tumor was removed completely. Intraoperative tumor perforation (intentionally or unintentionally) occurred in 34 patients. Uncontrolled blood exudation from the presacral venous plexus intraoperatively occurred in 2 patients, and a long piece of packing gauze was temporarily placed into the presacral space to achieve hemostasis. Seventy-two hours later, the packing gauze was removed. Cerebrospinal spinal leakage occurred intraoperatively in 1 patient, and a neurosurgeon was involved to manage it.

Of the 110 patients, 4 experienced complications after the operation. Wound infection occurred in 2 patients (grade I). After conservative treatment, the 2 patients achieved complete recovery. An anal fistula occurred in 1 patient (grade III) and required a second surgery. Postoperative cerebrospinal spinal leakage occurred in 1 patient (grade II) and was managed with conservative treatment after the operation.

All the other patients had uneventful recoveries and were safely discharged; no perioperative deaths occurred.

### Long-Term Follow-Up

Two patients with presacral adenocarcinomas and presacral stromal tumors in our series were lost to follow-up after the operation. The mean follow-up time was 90.13 ± 31.22 (range, 35-155) months.

By October 2021, 11 patients had local presacral tumor recurrence, and 1 patient had distant metastases, with a combined recurrence rate of 10.9% (n=12/110). None of the remaining patients (n=96) with presacral masses showed recurrence. Chronic sacral caudal pain occurred in 1 patient after resection of a presacral tumor, and constipation and loose stools occurred in 3 patients each after the operation.

Eight out of the 97 benign tumors showed recurrence (8.2%, n=8/97), whereas 4 out of the 11 consecutive malignant tumors showed local recurrence or distant metastases (36.4%, n=4/11). Presacral malignant tumors had a higher recurrence rate than presacral benign tumors (P< 0.05).

Among the 12 patients with recurrences or metastases, 1 patient underwent reoperation and radiotherapy, 2 patients underwent reoperation, and 6 patients are currently under observation for no obvious symptoms. One patient with a malignant teratoma died of tumor recurrence without reoperation, 1 patient with a carcinosarcoma died of metastases without reoperation, and 1 patient with fibromatosis died of recurrence after reoperation.

## Discussion

Here, we report our experience with the surgical resection of primary presacral tumors *via* a transsacrococcygeal transverse incision in terms of the surgical technique and long-term follow-up outcomes in a large sample.

According to the literature, presacral tumors occur more often in female patients than male patients (2, 9, 20). However, the reason for the female predominance among patients with presacral tumors remains unclear. One explanation is that the female predominance among patients with presacral tumors might be caused by selection bias because young females of childbearing age might undergo far more rectal palpations than young male patients (21). However, more research is needed to clarify the reason for the female predominance among patients with presacral tumors.

Presacral tumors are clinically rare entities with no specific symptoms. It is important to make a correct diagnosis of presacral tumors before surgery, but this diagnosis is not easy. In fact, a study by Singer et al. (22) showed that patients underwent an average of 4.1 surgical procedures before being correctly diagnosed with a primary presacral pathology. Previous studies have shown that sigmoidoscopy (23), proctoscopy (24) and endorectal ultrasound (15) can be utilized in the diagnostic process for presacral tumors. However, CT and MRI, with high sensitivity and specificity, are commonly used diagnostic methods in the treatment of presacral tumors. Using MRI and CT appropriately can improve the diagnosis rate of presacral tumors and can also help surgeons make optimal surgery plans. CT and MRI have become the standard diagnostic methods for presacral tumors (24).

The function of fine-needle biopsy in the treatment of presacral tumors remains controversial due to concerns about presacral hemorrhage and tumor spread *via* the needle tract. Previous studies showed that needle biopsy could increase the risk of infection of presacral tumors (25) or malignant changes in the teratoma (26). Most of the patients in our study did not undergo needle biopsy before surgery. Additionally, preoperative fine-needle biopsy does not affect the decision of surgery (7). However, some researchers (12, 27) have also suggested that for some solid presacral tumors, such as Ewin’s sarcoma, chordoma and lymphoma, preoperative fine-needle biopsy can help obtain pathological diagnoses, which may lead to benefits from chemotherapy and/or radiotherapy. Merchea et al. (28) showed that the preoperative fine-needle biopsy of solid presacral tumors was safe and that the results were highly concordant with those of postoperative pathology in comparison with imaging, with a sensitivity, specificity, and positive and negative predictive values of 96%, 100%, 100%, and 98%, respectively, for biopsy in detecting malignant disease. These results suggest that the preoperative biopsy of solid presacral tumors should be performed to guide subsequent treatment. However, the overall preoperative biopsy rates in published studies are low (8, 10, 23, 29).

A suitable surgical approach is an important factor for the successful resection of a presacral tumor. To make it easier to resect presacral tumors, surgeons have tried many approaches, including anterior, posterior, and combined approaches and laparoscopic surgery. After searching the literature, posterior approaches were determined to be the most common. When can a posterior approach be chosen for a presacral tumor? For the resection of presacral tumors, the literature showed that anterior approaches were performed when the lower border of the lesion was above the S3 level and that posterior approaches were chosen when the upper border was below the S3 level (23). However, this is not absolute. In addition, it has been reported that even if the presacral tumor reaches the S1 level, a posterior approach can also be used to achieve complete tumor resection (12). A previous study showed that if the upper border of the presacral tumor can be palpated by rectal palpation, a posterior approach can be chosen (22). However, we think that this notion is slightly conservative. In our experience, if half of the presacral mass can be palpated, it is likely that the presacral mass can be resected *via* a posterior approach by placing appropriate tension on the presacral tumor, which is consistent with the findings reported by Gordon PH (30).

There are many variations of posterior approaches, of which the Kraske and modified Kraske approaches are the most widely used. However, at our center, we prefer to apply a transsacrococcygeal transverse incision given the safety of the procedure. First, like all the other posterior techniques, the advantage of resecting presacral tumors *via* a transsacrococcygeal transverse incision is that it does not require accessing the abdominal cavity, so it can avoid a series of postoperative complications associated with open surgery, such as adhesive ileus. Second, compared to the Kraske incision, the transsacrococcygeal transverse incision allows the surgeon to use rectal palpation from the left and right sides to better expose the posterior wall of the rectum and then protect the rectum. Third, in most instances of procedures performed *via* a transsacrococcygeal transverse incision, the external sphincter muscle of the anus is not touched and is better protected, thereby better protecting the function of the anus.

An obvious concern is that the entire transsacrococcygeal transverse incision is close to the anus, which is prone to infection. To avoid wound infection, we employ the following practice: first, a drainage tube with a negative-pressure aspiration device is placed in the surgical site to fully drain the surgical site (**Figure 2**); second, the dressings are changed in a timely manner; and third, antibiotics are administered appropriately (anti-anaerobic bacterial drugs (metronidazole or ornidazole, etc.) + third-generation cephalosporins (ceftazidime or ceftriaxone, etc.). In our series, wound infection occurred in 1.8% of the patients (n=2/110). After conservative treatment, the 2 patients achieved complete recovery.

Because presacral tumors often compress the posterior wall of the rectum, it is occasionally difficult to resect presacral tumors completely without causing damage to the rectum. Rectal leakage, presacral infections and anal fistulas can arise as a result of injury to the rectal wall. In the case of rectal rupture, rectal repair can occasionally solve the problem. In our study, the posterior rectal wall was damaged during surgery and repaired after the presacral tumor was removed in 5 female patients. All of the patients recovered well, and none of them developed a presacral infection or an anal fistula. However, when the rectal rupture was large and the patient’s general condition was poor, a prophylactic colostomy (usually transverse colostomy or sigmoidostomy) was chosen to avoid rectal leakage and presacral infection. In our study, sigmoidostomy was performed in 1 male patient due to intraoperative rectal injury, and transverse colostomy was performed in 1 female patient due to intraoperative rectal injury.

During the operation, we inevitably encountered bursting of the cyst wall, which led to spillover of the bean curd residue-like (sebaceous) material in the cyst. The spillover can contaminate the surgical field. However, if we removed the cyst wall completely, rinsed the contaminant out of the surgical field completely, kept the drainage tube unblocked and changed the dressings regularly, the surgical incision could heal well, and tumor recurrence could be avoided. However, when the presacral cystic lesions were large, we intentionally elicited cystic fluids to reduce the tumor volume, which made it easy to treat the presacral lesions completely.

Surgeons who perform presacral tumor operations must be well aware that during surgery, they can face a difficult problem: uncontrolled intraoperative blood exudation from the presacral venous plexus. Uncontrolled intraoperative hemorrhage occurred in 2 patients in our study. To address this difficult problem, a long piece of packing gauze was temporarily placed in the presacral space to achieve hemostasis. Seventy-two hours later, when the hemorrhaging stopped, the gauze was removed, and the incision was sutured. Based on our experience, if properly handled, the use of gauze packing to stop uncontrolled presacral venous plexus blood exudation does not increase the rate of incisional infection. In our series, the surgical incisions of the 2 patients who were treated with gauze packing to stop presacral bleeding recovered well. However, more cases are needed to validate our research.

To better expose the surgical site, we occasionally had to resect the coccyx or even partially resect the sacrum. According to Shafik, removal of the coccyx and disconnection of the anococcygeal ligament do not affect the function of the anus (31). In our study, when necessary, a transsacrococcygeal transverse incision allowed the surgeon to dissect the coccyx, which helped to obtain sufficient exposure. In addition, removal of the coccyx reduces the recurrence rate because the totipotential cellular remnants in the coccyx are removed (32). However, other studies (2, 33) demonstrated that coccygectomy increased the mortality rate. When the presacral tumor is large, the sacrum (highest S3 level) can be removed to facilitate excision of the sacral tumor. However, the more sacrum that was removed, the more complications were observed after surgery (34). Previous studies have shown that to maintain normal urinary and fecal function, at least one or both S3 nerves must be preserved (27, 35). In our study of 110 surgeries, coccygectomy was performed in 35.5% of the patients (n=39/110), and coccygectomy plus partial sacrectomy was performed in 4.5% of the patients (n=5/110) (**Table 1**). Therefore, we suggest that if coccygectomy and sacrectomy facilitate the en bloc resection of presacral masses, the coccyx and sacrum should not be preserved.

In our study, the intraoperative complication rate was 42.7% (n=47/110), while the postoperative morbidity rate was 3.6% (n=4/110). In a multicenter French study (10), the intraoperative complication rate was 46% (n=124/270), and the postoperative morbidity rate was 30% (n=81/270). The difference in the intraoperative complication rate between our study and the French multicentric study was not statistically significant (P=0.57), whereas the postoperative morbidity rate in our study was lower than that in the French study (P<0.05) because more wound and pelvic infections occurred in the French study, which increased the postoperative morbidity rate. We think this difference may be explained by the low proportion of obese patients in our study. Obese patients are more likely to have wound infections after surgery. In our sample of patients, obese patients (BMI>28.0) accounted for a low proportion (8.2%, n=9/110) of the total, which may contribute to the low wound infection rate compared to the French study (**Table 1**).

The reported rates of presacral tumor recurrence after surgery in previous studies were 7.4% (10), 10.8% (11), 15.6% (24) and 20.4% (27). In our study, the recurrence rate was 10.9% (n=12/110). The lower postoperative morbidity rate, comparable intraoperative complication rate and recurrence rate in our study confirmed the feasibility and safety of the surgical resection of presacral tumors *via* a transsacrococcygeal transverse incision.

The length of hospital stay for the posterior approach was shorter than that for the anterior and combined approaches (13.3 ± 6.14 d vs. 19.0 ± 11.8 d), but the difference between the two groups was not statistically significant (P=0.056). We think this finding was correlated with the small number of patients treated with an anterior or combined approach, and more efforts are needed to further confirm the difference in the length of hospital stay between the two groups.

In conclusion, the surgical resection of primary presacral tumors *via* a transsacrococcygeal transverse incision is feasible and safe, with acceptable oncological therapeutic outcomes and a low postoperative morbidity rate, making it worth popularizing in clinical practice.

## Data Availability Statement

The original contributions presented in the study are included in the article/supplementary material. Further inquiries can be directed to the corresponding author.

## Ethics Statement

The studies involving human participants were reviewed and approved by the ethics committee of the Chinese PLA General Hospital. The patients/participants provided their written informed consent to participate in this study.

## Author Contributions

XZ and SZ conceived, designed the study and drafted the article. NL organized the database and performed the statistical analysis. PL and LC revised the paper critically and approved the final version of the paper. All authors contributed to manuscript revision, read, and approved the submitted version.

## Conflict of Interest

The authors declare that the research was conducted in the absence of any commercial or financial relationships that could be construed as a potential conflict of interest.

## Publisher’s Note

All claims expressed in this article are solely those of the authors and do not necessarily represent those of their affiliated organizations, or those of the publisher, the editors and the reviewers. Any product that may be evaluated in this article, or claim that may be made by its manufacturer, is not guaranteed or endorsed by the publisher.
